# Can a second resection be avoided after initial thulium laser endoscopic en bloc resection for non-muscle invasive bladder cancer? A retrospective single-center study of 251 patients

**DOI:** 10.1186/s12894-020-00599-1

**Published:** 2020-03-18

**Authors:** Wenhao Zhou, Wei Wang, Wenbo Wu, Tingmang Yan, Guofang Du, Haitao Liu

**Affiliations:** 1 Department of Urology, Shanghai General Hospital, Shanghai Jiao Tong University School of Medicine, No.100, Haining Road, Shanghai, 200080 China; 2 Weifang Second People’s Hospital, No.7 YuanXiao Street, Kuiwen District, WeiFang City, 261041 ShanDong Province China

**Keywords:** Intravesical instillation, Bladder cancer, Pirarubicin, Second resection, Transurethral thulium laser en bloc resection of bladder tumor

## Abstract

**Background:**

This study aimed to evaluate the efficacy of transurethral thulium laser en bloc resection of the bladder tumor (TmLRBT) in patients with non-muscle invasive bladder cancer (NMIBC) and to investigate whether a second resection can be avoided.

**Methods:**

From June 2012 to June 2018, 251 newly diagnosed patients with NMIBC were enrolled in this retrospective study; all patients received regular administration of pirarubicin after the initial resection. A second transurethral resection (TUR) was performed in patients within 2–6 weeks after the initial TmLRBT in group 1. Patients in group 2 only underwent cystoscopy at 3 months.

**Results:**

Second surgery results indicate that recurrence was detected histopathologically in 6/108 and 11/143 patients in group 1 and 2, respectively (*P* = 0.52); Progression was observed in 2 patients in each group (*P* = 0.34). The mean follow-up duration was 40.1 months, with no significant difference between the groups (*P* = 0.32). Recurrence was observed in 23 (21.3%) and 39 (27.3%) patients in groups 1 and 2 during the follow-up, respectively (*P* = 0.34); disease progression occurred in 4 (3.8%) patients in group 1 compared with 7 (4.0%) in group 2 (*P* = 0.20).

**Conclusion:**

Complete removal of tumors can be achieved by TmLRBT. This technique may decrease the number of second TURs.

## Background

Transurethral resection of the bladder tumor (TURBT) followed by intravesical instillation represents the gold standard for the treatment of non-muscle invasive bladder cancer (NMIBC). Ideally, all visible tumors can be removed and adequate specimens can be harvested by transurethral resection (TUR). This technique helps render the correct diagnosis and prevent early tumor recurrence and progression, thereby conferring a good prognosis [1]. However, the standard TUR involves piecemeal resection of the bladder tumor, which is associated with an elevated risk of tumor recurrence. Moreover, it is difficult to provide accurate pathological staging [2]. As for a second TUR, which is mandatory in cases of incomplete first resection, is currently recommended for high-grade Ta (TaHG) tumors and all T1 tumors [1], staging errors may be corrected and residual tumors can be detected [3], although it may lead to a significantly increased risk of complications and higher costs, which, to some degree, denotes the failure of the initial resection [4]. With the help of new techniques, a second TUR can be avoided and the overall treatment costs can be reduced.

Transurethral thulium laser en bloc resection of the bladder tumor (TmLRBT) is a new strategy for the treatment of NMIBC. Recent studies have shown that as a simple and reliable method, TmLRBT can lower the rate of perioperative complications and make the pathologic evaluations of specimens easier [5]. The presence of the detrusor muscle (DM) serves as a surrogate marker of resection quality, and TmLRBT can result in a higher DM rate (theoretically up to 100%) [6–8]. Kramer et al. [9] showed that the DM is found in 97.3% of patients who underwent en bloc resection of bladder tumors (ERBT). In some studies, the presence of the DM is an important parameter associated with recurrence and offers the possibility of reducing the need for a second resection [5, 6, 10, 11].

Herein, we aimed to evaluate the efficacy of TmLRBT in patients with NMIBC, and to investigate whether a second resection can be avoided by this means.

## Methods

### Patients

From June 2012 to June 2018, 251 patients with newly diagnosed NMIBC were selected for inclusion in this retrospective study. Patients were included if (1) imaging examinations showed that the bladder muscle had not been involved and there was no lymph node metastasis or distant metastasis; (2) they had normal functions of the main visceral organs (heart, liver, lungs, and kidneys); and (3) they needed a second TUR pursuant to the European Association of Urology guidelines, including those with T1 tumors and all HG/G3 tumors, excluding those with primary carcinoma in situ. Patients were excluded from the study if (1) distant metastasis, infiltration of the surrounding organs, metastatic bladder cancer, or other cancers involved the bladder; (2) the resection was incomplete because there was a lack of muscle tissues in the specimens; or (3) a muscle-invasive bladder tumor, carcinoma in situ, and upper urinary tract tumor were identified.

Patients were divided into two groups. Patients who had received a second resection at 2–6 weeks after the first resection were assigned to group 1. As for group 2, a second TUR was not performed at 2–6 weeks; instead, patients in this group underwent cystoscopy at 3 months.

### Data collection

The initial resection was performed in all patients by experienced urologists in our department with the thulium laser (2-μm continuous wave laser). The location, size, and number of tumors after the initial resection documented by the surgeon were recorded. The specimen should include the visible tumor, tumor base, and tumor margin.

### Surgical procedures

**Fig. 1:**
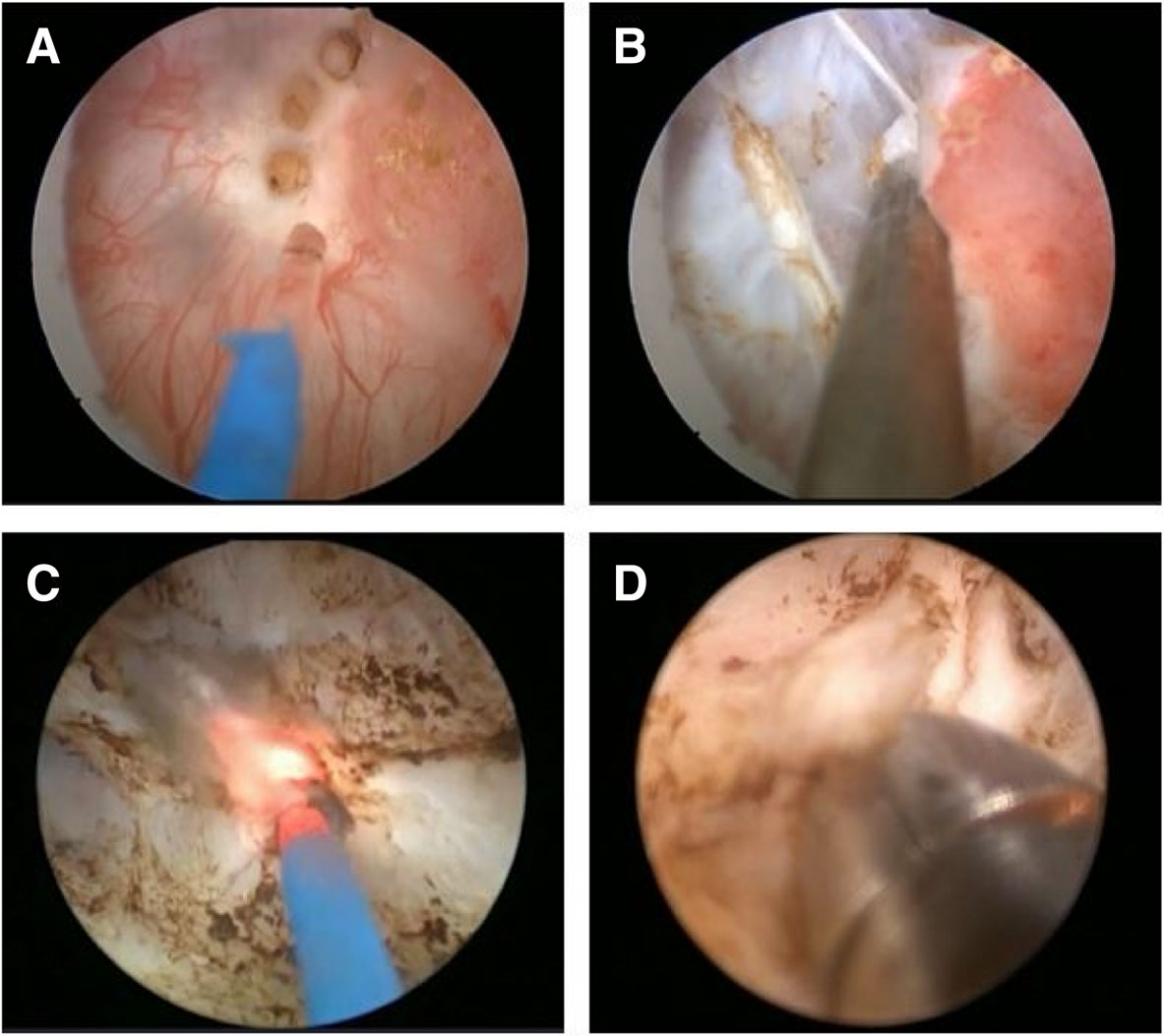
Transurethral thulium laser en bloc resection of a bladder tumor. **a** Circular incision around the tumor, **b** exposure of the tumor base via blunt dissection, **c** coagulation of the base of resection, and **d** cold-cup biopsy

TmLRBT was performed using the 2-μm thulium laser system (LISA Laser Products OHG, Katlenburg-Lindau, Germany) in continuous-wave mode. After continuous epidural anesthesia was administered, the patients were placed in the lithotomy position during the surgical procedure (Fig. 1a-d); the power of the laser was set at 30 W in advance, and the energy was delivered via a 550-mm end-firing PercuFib fiber (LISA Laser Products OHG) through a 26-French continuous flow resectoscope.

First, resection was performed along the peripheral marking line made approximately 1–2 cm outward from the tumor edge, and a fan-shaped incision, made from one side of the tumor base, proceeded along the marking line toward a greater range and depth of the muscle layer until the fibrous connective tissue of the outer layer of the bladder was exposed. Subsequently, we used the tip of the resectoscope sheath combined with the vaporization of the thulium laser to push the tumor base until the submucosa was exposed. Then, blunt dissection was performed along the loose space between the muscular layer and the connective tissue layer, and the muscle fiber was cut from the tumor base if they were connected to each other so that ERBT could be achieved. Saline irrigation was provided throughout the procedure, and the fiber tip should contact the tumor tissue precisely to avoid thermal damage.

In view of the number, size, and location of the bladder tumor, careful examinations before the resection were necessitated. When the tumor was single in number and measured < 3 cm, a complete tumor sample was removed; if the tumor measured > 3 cm, it was necessary to incise them into 2 or more portions longitudinally and/or along the tumor base. The specimens were extracted using an Elik evacuator and sent to the pathological department for diagnoses. Tumors were classified according to the 2009 Union for International Cancer Control TNM system, and histopathology was performed according to the 1973 and 2004 World Health Organization grading system classification. All patients were catheterized postoperatively. The Clavien-Dindo classification of complications was adopted.

### Adjuvant intravesical chemotherapy

After indwelling the catheter, bladder irrigation with pirarubicin (50 mg/50 ml) was started and maintained for 15 min. Then, it was administered on a weekly basis for 8 weeks starting from 1 week postoperatively, followed by monthly maintenance until 12 months.

### Follow-up

All patients in group 1 underwent a second resection within 2–6 weeks, and all patients in group 2 underwent cystoscopy at 90 days after the initial TmLRBT. The presence or absence of tumors and the tumor characteristics were evaluated, and the pathological examination of the specimens was conducted by the same pathologists in our hospital. Follow-up was performed with ultrasonography, urinary cytology, and cystoscopy at an interval of 3 months during the first 2 years, and interval of 6 months during the next 3 years.

### Statistical analysis

The data are expressed as mean ± standard deviation. The data were analyzed using the χ^2^ test, and data were compared between the two groups using the *t* test. A *P*-value < 0.05 indicated a significant difference. Statistical analysis was performed using the JMP 10.0.0 software (SAS Institute, Cary, NC, USA).

## Results

Two hundred fifty-one patients were enrolled in this study, including 108 in group 1 and 143 in group 2. The characteristics of patients and tumors at the time of initial resection are shown in Table 1. According to the Clavien-Dindo classification of surgical complications, no intraoperative or postoperative complications developed in the patients. In addition, the bladder DM was present in all specimens.

**Table 1:** Characteristics of patients and tumors at the initial resection

Variable	Group 1	Group 2	*P*-value
Female sex (n, %)	22 (20.37)	32 (23.38)	0.70
Male sex (n, %)	86 (79.63)	111 (76.62)	
Mean age (years)	66.12 ± 1.52	68.59 ± 1.36	0.49
Number of tumors	2.63 ± 0.24	2.61 ± 0.22	
1 (n, %)	56 (51.85)	74 (51.75)	0.95
2–5 (n, %)	38 (35.19)	50 (34.97)	
> 5 (n, %)	14 (12.96)	19 (13.28)	
Mean diameter of the neoplasms (cm)	2.74 ± 0.13	2.49 ± 0.11	0.10
Stage (n, %)			
Ta	60 (55.56)	87 (60.84)	0.40
T1	48 (44.44)	56 (39.16)	
Grade (2004)^a^ (n, %)			
Low-grade	25 (23.15)	49 (34.27)	0.06
High-grade	83 (76.85)	94 (65.73)	

Data are displayed as n or mean ± standard deviation (range)

^a^ World Health Organization classifications

All patients underwent a second surgery and cold-cup biopsy, which included the tumor base and new neoplasms, and the results of the second resection are shown in Table 2. In group 1, the postoperative pathological examination revealed recurrence in 6 (5.6%) patients and disease progression in 2 (1.9%) patients. In group 2, the postoperative pathological examination showed recurrence in 11 (7.7%) patients and disease progression in 2 (1.4%) patients. Recurrence and progression occurred in patients with stage T1 tumors in both groups, but recurrence and progression did not occur in patients with TaHG tumors. The tumor-free status of all the remaining patients was histologically confirmed at the time of the second resection.

**Table 2:** Results of the second surgery

	Second Resection	Cystoscopy at 3 Months	*P*-value
Pathological results	108	143	
Relapse	6 (5.56)	11 (7.69)	0.52
Disease progression	2 (1.85)	2 (1.40)	0.34

Data displayed as n (%)

**Fig. 2:**
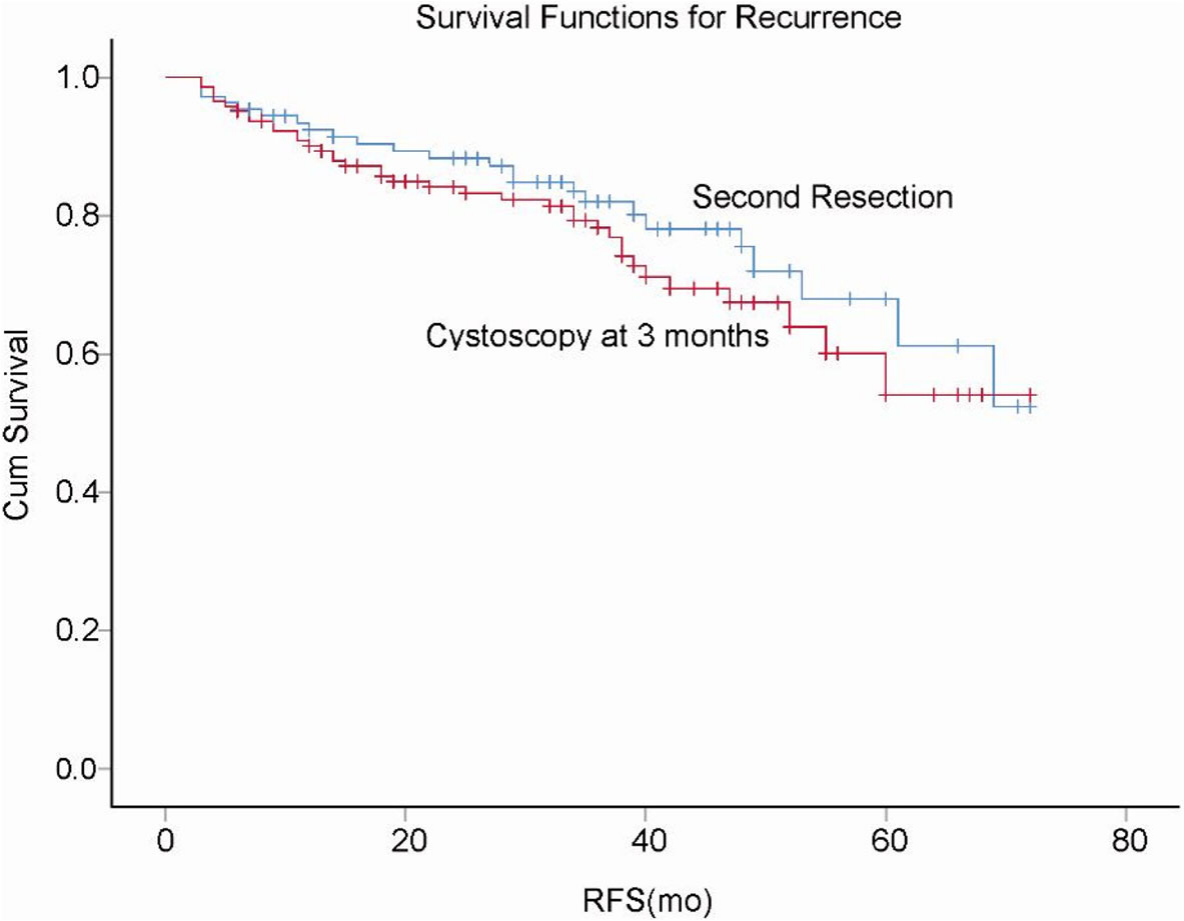
Kaplan-Meier curve showing recurrence-free survival (RFS) rates between the two groups (log-rank test result: 0.287)

After a median follow-up of 40.1 months (range, 3–72 months), the average duration of follow-up between the two groups was not significantly different (group 1, 41.5 months; group 2, 39 months; *P* = 0.32). Four and 9 patients were lost to follow-up in group 1 and group 2, respectively, with a loss rate of 5.2%. Recurrence and progression occurred in 23 (21.3%) and 4 (3.8%) patients, respectively, in group 1 during the follow-up, as compared with 39 (27.3%) and 7 (4.0%) patients, respectively, in group 2. The recurrence-free curves for the two groups are shown in Fig. 2. The recurrence-free survival rates in groups 1 and 2 were 92.6 and 90.2%, respectively, in the first year, and those were 84.3 and 80.4%, respectively, in the third year. The progression-free survival rates were 98.1 and 97.9%, respectively, in groups 1 and 2 in the first year, and those were 96.3 and 95.1%, respectively, in the third year. There were no significant differences in the rates of both recurrence and progression between the two groups.

## Discussion

The goal of TURB is not only to remove all visible lesions but also to offer adequate specimens, whereby a correct diagnosis can be made [1]. However, the high rate of residual tumors and the frequently understaged tumor stage at a second resection after the initial TURBT often make it impossible to attain such a goal [4]. The skills of surgeons, availability of newer endoscopes, presence or absence of the DM in the first specimens, and intravesical chemotherapy are all factors that affect the results, and the rates of residual tumors and recurrence have remained unacceptably and constantly high over the last 2 decades [12, 13]. As revealed in the studies by Herr in both 1999 and 2005, the rates of residual tumors were 75 and 15% to 53%, respectively, at second resection [14, 15]. To solve this problem, many urologists have now confirmed that a second resection or regularly repeated resection can be employed to detect residual tumors and improve staging, thereby increasing recurrence-free survival and reducing tumor burden [1, 3, 16, 17].

We believe that a second TUR after a complete TUR is associated with a significantly decreased rate of recurrence and progression in patients with newly diagnosed NMIBC. As shown in Divrik et al.’s study, even if the primary TUR was complete and the muscle tissue was present in all specimens, persistent diseases can still occur in one-third of patients with T1 bladder tumors [16, 17]. Overemphasis of the role of a second resection may mean, in a sense, failure of the technique [2]. It has been long demonstrated that TURBT does affect the volume of viable tumor cells released during the tumor resection in animals [18]. To ensure that complete resection is performed and the correct diagnosis is rendered, various en bloc resection techniques have been applied in several studies, e.g., the modified loop J-shaped needle electrode by Ukai et al. [19], holmium laser by Saito et al. for tumors at the bladder neck [20], and thulium laser by Wolters et al., who demonstrated the presence of DM in all cases [21]. In recent studies, Muto et al. and Migliari et al. confirmed that TmLRBT is a simple, reliable technique, and believed that the need for a second TUR might be reduced after TmLRBT in patients with NMIBC [5, 22].

In our early experience, we used the thulium laser for the treatment of benign prostatic hyperplasia, and its high efficacy and low perioperative morbidity have been proven [23]. Subsequently, Liu et al. [24] and Zhong et al. [25] found that compared with TURBT and the holmium laser, a combination of TmLRBT and intravesical chemotherapy scarcely resulted in bleeding and perioperative complications in patients with multiple NMIBC, and that the rates of recurrence during the 24-month follow-up showed no statistical differences. However, Kramer et al. [26] revealed that the thulium laser can produce a smoother cutting line, and thus, a more accurate incision than the holmium laser. It seems that the thulium laser may represent an appropriate modality to achieve complete resection and make the correct diagnosis.

TmLRBT makes it possible to eliminate tumor remnants and achieve complete resection of tumors, although some studies reported that the rate of residual T1 tumors was 33–55% at a second resection, and the rate of TaHG tumors was about 41% [17, 27]. Analysis showed that TmLRBT has certain advantages in terms of complete resection of tumors for the following reasons: it is operationally simple, causes minor thermal damage, and generates no electric currents, effectively overcoming obturator nerve reflex and bladder perforation. Moreover, it can demonstrate a good hemostatic effect, produce clear surgical fields of vision, and remove the primary tumors and minor lesions in a timely manner.

Compared with TURBT alone, the addition of single immediate instillation of pirarubicin can reduce the rate of recurrence by 11.7 to 13.0% [1, 28], and chemotherapeutic agents, such as mitomycin C, epirubicin, and doxorubicin, have shown beneficial effects. In addition to bleeding, tumor cell implantation is a major perioperative complication, and immediate single instillation can destroy the disseminated and residual tumor cells [29]. In the present study, all patients received immediate single instillation with pirarubicin (50 mg/50 ml), and then they were instilled once weekly for 8 weeks, followed by maintenance once monthly until the twelfth month.

Through a secondary resection, surgeons can find residual tumors, and correct clinical staging and pathological grades; besides, this treatment option can prolong the recurrence-free survival of patients. In a prospective, randomized, controlled study [30], patients with T1 tumors were divided into a secondary resection group and non-secondary resection group at the initial diagnosis, and the average follow-up duration was 66.1 months (range, 12–102 months). The recurrence-free survival rates were 82 and 65%, respectively, in the secondary resection group during the first and third years, as compared with 57 and 37%, respectively, in the non-resection group, demonstrating that a secondary resection can significantly improve the recurrence-free survival rate of patients. There were no statistically significant differences between our study and that by Divrik et al. in terms of patients’ general conditions, and postoperative pathological grades and clinical stages. Although the rates of recurrence-free survival and recurrence were not significantly different between the two groups in our study, they were superior to the results of an initial TURBT plus a secondary resection, as reported in the literature.

The limitations of this study were mainly as follows. Firstly, this study was not buoyed by any multi-center, prospective, randomized, higher-level evidence-based studies. Secondly, the surgical procedures were changed. Although operations were performed by proficient senior surgeons, the surgical methods and steps were changed; therefore, the findings might be biased. Lastly, the information obtained from the long-term follow-up needs to be improved, and the impacts of non-secondary resection on the prognosis of tumors require further validation.

## Conclusions

TmLRBT can serve as a feasible and efficient option for the treatment of NMIBC. It can result in a decreased rate of perioperative and postoperative complications. Moreover, complete removal of the tumor tissue can be achieved using TmLRBT, and it can provide adequate specimens for pathological grading and clinical staging. This may decrease the number of second TURs. Compared with patients who have undergone second TUR, those who have not were not statistically different in short-term tumor recurrence and progression; therefore, conclusions regarding whether a second resection can be avoided warrant long-term follow-up and clinical studies with a large sample size.

## Data Availability

The datasets analyzed during the current study available from the corresponding author on reasonable request.

## Abbreviations

TURBT: Transurethral resection of the bladder tumor
NMIBC: Non-muscle invasive bladder cancer
TUR: Transurethral resection
DM: Detrusor muscle
ERBT: En bloc resection of bladder tumors
TmLRBT: Transurethral thulium laser en bloc resection of the bladder tumor
